# Liquid Chromatography Mass Spectrometry Quantification of α-solanine, α-chaconine, and Solanidine in Potato Protein Isolates

**DOI:** 10.3390/foods9040416

**Published:** 2020-04-02

**Authors:** Søren D. Nielsen, Jesper M. Schmidt, Gitte H. Kristiansen, Trine K. Dalsgaard, Lotte B. Larsen

**Affiliations:** 1 Department of Food Science, Aarhus University, Agro Food Park 48, DK-8200 Aarhus N, Denmark; jesper@food.au.dk (J.M.S.); ghk@food.au.dk (G.H.K.); trine.dalsgaard@food.au.dk (T.K.D.); lbl@food.au.dk (L.B.L.); 2 iFOOD Aarhus University Centre for Innovative Food Research, Aarhus University, DK-8200 Aarhus N, Denmark

**Keywords:** glycoalkaloid, single ion monitoring, potato protein, plant protein, sustainability

## Abstract

For potato proteins to be used as a food ingredient, the level of natural potato defense substances, the glycoalkaloids (GAs), should be limited. In this work, a method is developed for quantification of the two major potato GAs, α-solanine and α-chaconine, as well as for their aglycon form, solanidine, using liquid chromatography–mass spectrometry single quadrupole in single ion monitoring mode. Standard solutions of GA and a food-grade potato protein powder was used to validate the method. A linear correlation between GA concentration and the ion intensity of >0.995 was obtained for all standard solutions. Recovery of GA in spiked samples was within the range 82%–106%. The method for GA quantification was applied to a variety of potato protein isolates. The results showed that total GA increased during the storage of the potatoes. Washing the potato protein isolates using water at a sufficient level was shown to be able to reduce the amount of GA below the threshold of 150 µg g^−1^, as needed for human consumption.

## 1. Introduction

Alternative sources for dietary proteins, and, not the least, for vegetable proteins, are of major current interest. Using the side-product (potato fruit juice) from potato starch production represents a sustainable use of a side-stream from other food production and is of interest to the industry as an additional value creation. The potato fruit juice contains approximately 2%–5% solid material, of which 35% is crude protein, giving a crude protein content of 1%–2% [1]. The proteins can be divided into three major groups: patatin (40% of total protein), protease inhibitors (50% of total protein), and other proteins (10% of total protein) [2]. The protein has a high nutritional value and good functional properties and could be used as an ingredient in food products [3,4]. When purifying the protein from potato fruit juice, the concentration of glycoalkaloids (GA) normally increases, as GA is purified together with the proteins. However, before potato protein can be used as an ingredient in the food industry, it is needed that the levels of the toxic and bitter-tasting potato GAs are reduced. The presence of GAs has furthermore been shown to result in lower consumer acceptability [5]. The two major GA in potatoes are α-solanine and α-chaconine, both consisting of the alkaloid solanidine, bound to a trisaccharide. The structure of the trisaccharide differs; α-solanine is a β-solatriose (α-L-rhamnopyranosyl-β-D-glucopyranosyl-β-galactopyranose), while α-chaconine is a β-chacotriose (bis-α-L-rhamnopyranosyl-β-D-glucopyranose [6]. The tri-glycoalakloid (α-compound) can be hydrolyzed by enzymes or acid treatment, yielding two different di-glycoalkaloids (β-compounds), one mono-glycoalkaloid (γ-compound), and the aglycon structure [6]. The concentrations of GAs is highest in the blossoms (3000–5000 mg/kg) compared to the sprouts (2000–4000 mg/kg) and peel (300–600 mg/kg), whereas the average level across the entire potato tubers is approximately 100 mg/kg [7]. Furthermore, the level of GAs increases during the storage of potatoes after harvest [8].

The toxic GAs function as a natural defense towards infectious agents within potatoes, and upon consumption, it may result in different symptoms that may include nausea, diarrhea, fever, and even death [6]. The underlying mechanism of GA toxicity is related to an inhibitory effect on the enzymes acetylcholinesterase and butyrylcholinesterase, both involved in the hydrolysis of the neurotransmitter acetylcholine [9,10]. Furthermore, it has been shown that GA interferes with ion-transport in cell membranes and can lead to membrane disruption [6]. Due to this toxicity, the European Commission decided that the total GA content must not exceed the limit of 150 µg g^−1^ in potato protein powder for food applications [11].

The GA content in potato protein isolates is tightly related to the isolation method. The traditional preparation method is isolation from potato fruit juice by heat coagulation, which results in denatured protein precipitate (especially patatins) with very low solubility, as well as co-precipitation of GA [12]. More gentle methods, such as ultrafiltration or chromatography, results in a native protein with high solubility, lower amounts of GA, and the possibility for further fractionation to purify specific protein fractions. Although these fractions potentially have lower amounts of GA, their content of solanidine has not previously been determined [12]. The state of the protein, native or denatured, affects protein solubility in the extraction solvents used for the analysis of GA content, with a high solubility for native protein and, furthermore, especially for the group of potato proteins known as the protease inhibitors (PI) [13]. For food grade protein isolates, the low levels of GA need to be determined with high precision, which is obtained by using ions from mass spectrometry detection for quantification. Previous methods to quantify potato GA used thin-layer chromatography [14], enzyme-linked immunosorbent assays [15], matrix-assisted laser desorption/ionization time of flight mass spectrometry [16], or chromatographic quantification using peak area of UV chromatograms [17], but methods based on mass spectrometry analysis have also been described, using untargeted [18] or targeted analysis [19]. Often analysis of potato protein isolates will involve initial solid-phase extraction methods, which will lead to co-extraction of other compounds. Using UV-based detection of GA and hence quantification based on peak area can be overestimated.

The aim of this study is to develop a method for absolute quantification of potato GA (α-solanine and α-chaconine) based on liquid chromatography–electrospray ionization mass spectrometry (LC–ESI/MS) in single ion monitoring (SIM) mode. The developed method also quantifies the alkaloid cleavage product (solanidine) from GA, which is often lacking in other studies and mainly measured by UV-based methods [20,21]. This method was applied to a number of potato protein isolates of relevance to the food industry. We investigated the role of chromatographic purification, removal of GA by washing with water, and the effect of storage time on GA level in protein from potato tubers following harvest. We also investigated two laboratory-produced potato protein isolates fractionated into a PI and a patatin fraction for their content of α-solanine, α-chaconine, and solanidine.

## 2. Materials and Methods

### 2.1. Materials

LC–MS grade acetonitrile (ACN), acetic acid, and LC–MS grade methanol was obtained from Merck (Kenilworth, NJ, USA). LC–MS grade formic acid (FA) was obtained from Honeywell Fluka (Roskilde, Denmark). Tomatine was purchased from Carlroth (Karlsruhe, Germany). α-solanine, α-chaconine, and solanidine were purchased from Sigma-Aldrich (St. Louis, MO, USA).

### 2.2. Potato Protein Samples

In total, seven different potato protein isolates were tested. Five of these were heat-denatured protein isolates produced from potato fruit juice as a waste product from potato starch production were provided by KMC, Brande, Denmark. Three samples were potato protein isolates produced for animal feed (feed grade protein) with different in-silo storage time after harvest from September to January. Two of the five heat-denatured protein isolates were extensively washed with water to lower the GA content into a grade suitable for food protein in either a high or low protein-to-water ratio. In addition, two laboratory-produced native protein isolates representing specific protein sub-fractions were included, fractionated from potato fruit juice by ion-exchange chromatography [22], representing patatin- and protease inhibitor (PI)-rich fractions. In brief, the purification procedure of the two protein sub-fractions was performed by adjusting potato fruit juice to pH 8, followed by anion exchange chromatography (DEAE Sepharose Fast Flow medium), dialysis, and concentration by ultrafiltration, and, finally, freeze-drying.

### 2.3. Sample Preparation

The extraction of potato GA was carried out using a previously published method [23], but with some modifications. Ten mg of potato protein isolate was added to 1980 µL 5% acetic acid and 20 µL internal standard (Tomatine, 30 µg mL^−1^ giving a final concentration of 0.3 µg mL^−1^) and shaken for 15 min. The sample was then centrifuged for 15 min (14,000× *g* at 4 °C), and the supernatant was collected for further purification. Extraction of potato GA was conducted by solid-phase extraction (SPE) cartridges (HLB Oasis 1cc 30mg, GE Healthcare) in a vacuum manifold. In general, the volume of solution used in each step was 1 mL and repeated three times, unless otherwise stated. The column was pre-conditioned with methanol, and then equilibrated with MilliQ water before 2 mL of each sample was loaded onto the column. The column was subsequently washed with 10% methanol, and, finally, the purified GA was eluted with 1 mL methanol containing 0.1% formic acid. The eluate was filtered through a 0.2 µm Mini-UniPrep filter (Whatman, Maidstone, United Kingdom) before injection onto the LC–ESI/MS.

The standard solutions for α-solanine, α-chaconine, solanidine, and tomatine were prepared by making a stock solution in methanol (500 µg mL^−1^) and stored at −80 °C. Working standard solutions were prepared by dilution of the stock solution in methanol. Chemical structures of glycoalalkaloid were drawn with ChemSpider (www.chemspider.com).

### 2.4. Liquid Chromatography–Electrospray Ionization Mass Spectrometry Analysis

LC–ESI/MS was conducted to quantify α-solanine, α-chaconine, and solanidine. The samples were loaded onto a Kinetex C18 column (250 × 4.6 mm) with a particle size of 5 µm (Phenomenex, Torrance, CA, USA) on an Agilent 1260 Infinity II LC system. Solvent A was 0.1% FA, and solvent B was 100% acetonitrile and 0.1% FA. The gradient started at 28% B and increased to 32% B over 11 min and then increased to 41% B over 1 min and to 45% B over 8 min. Finally, the gradient was increased to 90% B over 1 min and held at 90% B for 5 min, until returning to 28% B over 1 min. The MS fragmentor was set at 120 V. The flow rate was set to 0.5 mL/min, and the injection volume of the samples and standards was 5 µL.

The mass spectrometry analysis was carried out on an InfinityLab single-quad mass spectrometer (Agilent Technologies, Palo Alto, CA, USA). Electrospray ionization was performed in positive mode and scan mode conducted with a scan range of 50 to 1200 *m*/*z*. Absolute quantification of GA was conducted using selected ion monitoring mode (SIM, Table 1). For each compound, the highest ion was selected as the target ion, and the two second-highest signals were used as qualifier ions. For α-solanine, α-chaconine, and solanidine, the target ion was also the mother ion. The amount of GA was calculated as the integrated intensity of the target ion for each compound and compared to the external calibration curve of α-solanine and α-chaconine (0.03 to 3 µg mL^−1^) and of solanidine (0.03 to 1 µg mL^−1^). Variation during GA purification and instrument performance was normalized using tomatine as an internal standard (ISTD).

### 2.5. Data Analysis

Validation of the method was carried out using standard solutions of α-solanine, α-chaconine, and solanidine in accordance with the FDA guidelines. Accuracy is the relative error of the calculated concentration versus the expected concentration. Accuracy of inter-day and intra-day variation was calculated as

(1) $$\mathrm{Accuracy}=\frac{\mathrm{Calculated}\;\mathrm{Concentration}}{\mathrm{Expected}\;\mathrm{Concentration}}\times 100$$

Precision of inter-day and intra-day variation was calculated as the relative standard deviation:

(2) $$\mathrm{Precision}=\frac{\mathrm{Standard}\;\mathrm{deviation}}{\mathrm{Mean}}\times 100$$

Limit of detection (LOD) was calculated as

(3) $$\mathrm{LOD}=\frac{3.3\sigma }{b}$$

where *b* is the slope of the regression line, and *σ* is the standard deviation of the response. Lower limit of quantification (LOQ) was defined as the lowest level in the linearity range.

Statistical analysis was carried out in R using a one-way ANOVA test with Tukey’s post hoc test. GA level was compared within samples from either feed grade, food grade, or chromatography isolation. Significant difference was defined as *p* < 0.05.

## 3. Results and discussion

### 3.1. Liquid Chromatography–Electrospray Ionization Mass Spectrometry Single Quadrupole Analysis

**Figure 1:**
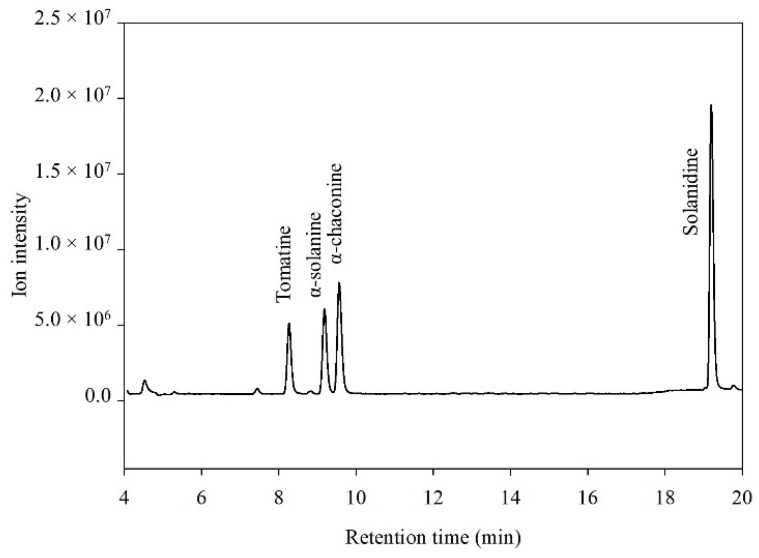
Liquid chromatography–electrospray ionization mass spectrometry (LC–ESI/MS) chromatogram in full scan mode of standard solutions of tomatine (10 µg mL^−1^), α-solanine (10 µg mL^−1^), α-chaconine (10 µg mL^−1^), and solanidine (10 µg mL^−1^).

**Figure 2:**
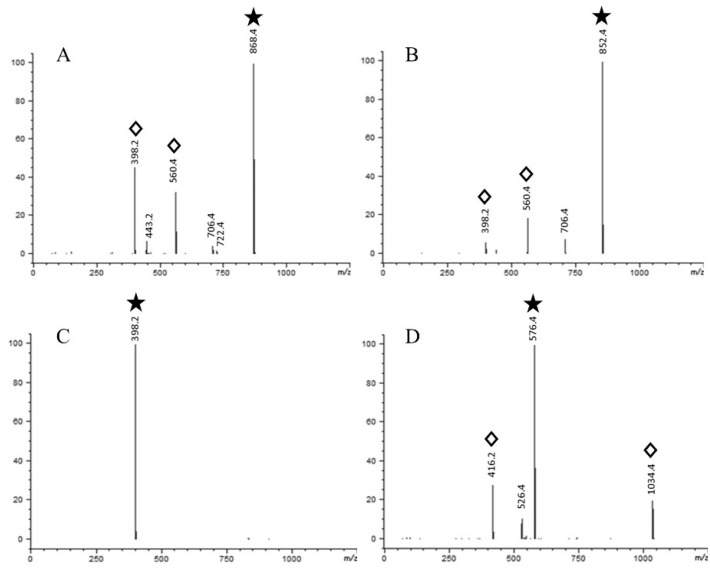
Mass spectrometric spectra of the standard solutions of α-solanine (**A**), α-chaconine (**B**), solanidine (**C**), and tomatine (**D**) at 10 µg mL^−1^ in scan mode. Fragment masses are shown above peaks. Diamonds above masses are ions used for target (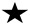) and qualifier ions in single ion monitoring mode (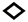).

**Figure 3:**
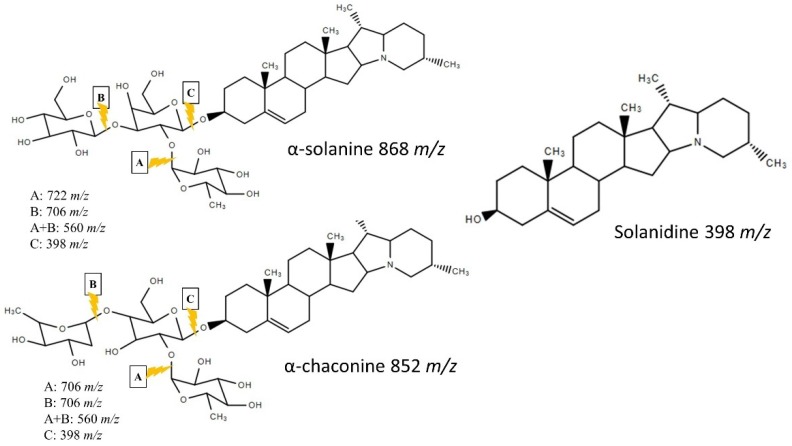
Structure of α-solanine, α-chaconine, and solanidine with indication of their fragmentation (drawn using ChemSpider). Lightning indicates in-source fragmentation producing addition ions.

The standards of tomatine (IS), α-solanine, α-chaconine, and solanidine were initially analyzed by LC–ESI/MS in scan mode (Figure 1). The retention time for tomatine was 8.1 min, 9.1 min for α-solanine, 9.4 min for α-chaconine, and 19.3 min for solanidine. Fragmentation ions of the compounds were identified and are shown in Figure 2. The masses of these fragments were in accordance with the release of one or more of the monosaccharides from the alkaloid structure (Figure 3). Fragmentation of α-solanine resulted in the release of β-D-glucose corresponding to a remaining alkaloid molecule with *m*/*z* of 706 or release of α-L-rhamnose corresponding to a remaining ion *m*/*z* 722, both occurring from cleavage of a single glycosidic bond and subsequent rearrangement of a hydrogen. Removal of both β-D-glucose and α-L-rhamnose parts resulted in the remaining ion with *m*/*z* of 560, while removal of the β-D-galactose also yielded the bare alkaloid, solanidine, with a fragmentation ion of *m*/*z* 398 (Figure 3). The fragmentation of α-chaconine corresponded to the removal of either of the two α-L-rhamnoses, resulting in the remaining ion with *m*/*z* of 706. Removal of the second α-L-rhamnose resulted in the ion *m*/*z* 560. Further removal of β-D-glucose yields solanidine (Figure 3). The aglycon solanidine did not fragment any further.

The most abundant ion from each compound was selected for quantification (target ion) and the additional two ions from tomatine, α-solanine, and α-chaconine were selected as qualifier ions. Our method is, therefore, highly specific as it uses three ions from each of these compounds to confirm that we quantified the correct compound. In contrast, solanidine was quantified based on a single specific ion as it did not fragment. The current state-the-art method for quantification using mass spectrometry is based on multiple reaction monitoring (MRM). MRM is, like SIM analysis, based on targeted analysis of a selected mass representing one or more specific molecules, but while MRM uses tandem mass spectrometry, SIM is possible using the cheaper and simpler LC–SI/MS single quadrupole system.

The most intense ions from each compound were selected for single ion monitoring and retention time windows set for each compound, as some of the fragment ions were identical between the compounds (Table 1). A linear correlation between GA concentration and the ion intensity for each compound was obtained in the range from 0.03 to 3 µg mL^−1^ for α-solanine (r^2^ = 0.994) and α-chaconine (r^2^ = 0.996) and from 0.01 to 1 µg mL^−1^ for solanidine (r^2^ = 0.997). Beyond these ranges, the curve was no longer linear. The limit of quantification was 0.03 µg mL^−1^ for α-solanine and α-chaconine and 0.01 µg mL^−1^ for solanidine. The limit of detection was 0.01 µg mL^−1^ for α-solanine and α-chaconine and 0.003 µg mL^−1^ for solanidine (Table 2). In multiple previous studies, UV signal has been used for quantification of GA content in potatoes; however, this is less sensitive than our MS-based method and will have a higher limit of quantification. In one study, the linear range of detection was found to be 2–17 µg mL^−1^ for chaconine and 2.5–28 µg mL^−1^ for solanine using HPLC equipped with a UV detector [24]. Another study found the linear range was 5 to 250 µg mL^−1^ using HPLC–UV/vis [23]. Laus and colleagues found that they could reliably quantify as low as 0.125 µg mL^−1^ [13].

Solid-phase extraction clean-up of potato protein powders resulted in higher signal intensities of the compounds compared to samples with no SPE clean-up (results not shown). Elution of GA from the SPE cartridges with 1 mL of methanol was sufficient for complete extraction, as additional elutions with 1 mL methanol from the SPE cartridges contained less than 0.01% of the GA contents measured in the first elution. For verification of the extraction method, two known concentrations (2 and 0.03 µg mL^−1^ of both α-solanine and α-chaconine, and 0.6 and 0.03 µg mL^−1^ of solanidine) was added to the potato protein powder dissolved in acetic acid solution and compared to an un-spiked sample. The recovery of α-solanine ranged from 81.6% to 106.4%; for α-chaconine, the recovery ranged from 82.7% to 101.5%, and for solanidine, the recovery ranged from 83.9% to 90.3%.

The intra-day precision was estimated with a coefficient of variance below ten for all three compounds, while the accuracy ranges from 86.4% to 114.3%. The inter-day precision was estimated to have a coefficient of variance below 11 and an accuracy ranging from 79.9% to 128.8% (Table 3), which is within an acceptable range.

### 3.2. Glycoalkaloids in Potato Protein Isolates

**Figure 4:**
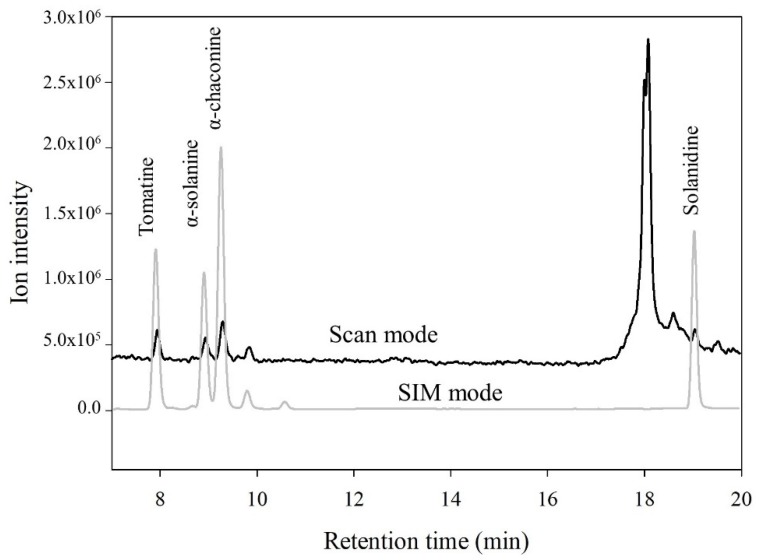
Identification of potato glycoalkaloids in food-grade potato protein powder by LC–ESI/MS in scan mode and single ion monitoring (SIM) mode.

All samples were investigated in both SIM and scan mode. In Figure 4, the total ion chromatogram from both a scan and SIM mode analysis of a food-grade potato protein isolate was investigated. The SIM mode resulted in a significantly higher response compared with scan mode—approximately 10-fold higher—indicating a much higher sensitivity due to increased dwell time of each ion. In the potato protein, sample the peak area of solanidine in SIM mode can be more precisely determined as it is baseline separated, while in scan mode, it is more difficult to estimate the peak area as it coelutes with other compounds (Figure 4).

**Figure 5:**
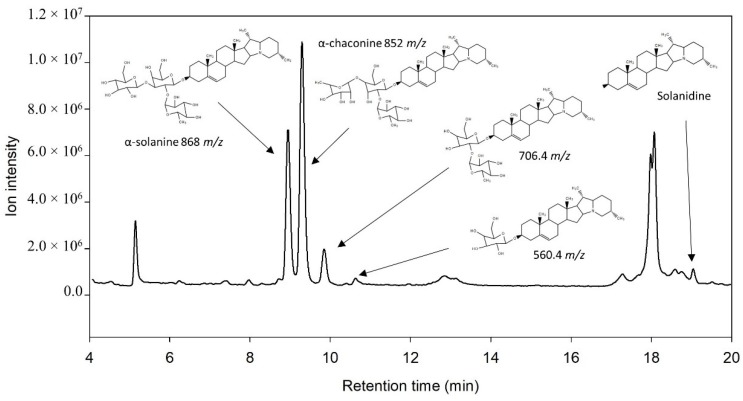
LC–ESI/MS chromatogram of feed-grade potato protein power and the different forms of glycoalkaloids identified.

The total ion chromatogram of a feed grade protein isolate in scan mode showed peaks of α-solanine, α-chaconine, and solanidine. Peaks containing the masses of 706.4 *m*/*z* and 560.4 *m*/*z* were also identified (Figure 5). The ion of 706.4 *m*/*z* likely represents β_1_-solanine, β_1_-chaconine, and β_2_-chaconine, which all have identical masses, and occur after removal of one of the two α-L-rhamnose from α-chaconine or removal of β-D-glucose from α-solanine [25]. The peak of 560.4 *m*/*z* likely represents γ-chaconine or γ-solanine. Only the β-D-glucose remains in γ-chaconine, while only β-D-galactose remains in γ-solanine.

The content of α-solanine, α-chaconine, and solanidine of the different potato protein powders are displayed in Table 4. Feed-grade protein isolate had a high total GA content, which correlated with the time of processing of the potatoes. The concentration of solanidine also increased during in silo storage. Those potatoes processed at a later timepoint had the highest levels of GA, which suggest that the conditions at which the potatoes are stored induce the level of GA in the potatoes. This confirms a previous result showing that prolonged storage under either indirect sunlight, dark room, dark room with cooling, or under fluorescent light were all associated with increased levels of GA in potatoes [8]. The concentration of solanidine increased as well during the storage of the potatoes.

Washing the denatured protein with water (representing a food-grade protein isolate) resulted in a significant reduction of both α-chaconine and α-solanine to the same extent, hence, decreasing the total GA content from being in the range 2061–3842 µg g^−1^ to 29–316 µg g^−1^ (current regulation requires a value below 150 µg g^−^^1^). The two food-grade proteins were washed at either high or low protein concentration, which highly impacted to what extend the total GA was removed from these samples. Potato GAs, in general, have low solubility in water at neutral pH, as observed by boiling potatoes, inducing a reduction of contents of α-chaconine or α-solanine by only 3.5% and 1.2%, respectively [26]. The solubility of potato GAs in water, however, will increase at lower pH values [27]. Methods to remove glycoalkaloids from potato protein are scarcely reported in scientific literature, and much knowledge is of proprietary status. Giuseppin and Spelbrink [28] reported that ultrafiltration and diafiltration can remove some glycoalkaloids; furthermore, acidic extraction or fermentation was suggested, as well as treatment with activated carbon to bind the glycoalkaloids. Marchal et al. [29] suggested the removal of glycoalkaloids by washing with acids, organic solvents, or a combination thereof. Backleh et al. [30] found that adsorptive bubble separation at pH 6 could be used to remove glycoalkaloids from potato protein solutions. Ralla et al. [31] reported the removal of glycoalkaloids by binding to clay particles during the purification of potato protein.

The laboratory-produced native potato protein fractions (PI and patatin) differ in GA content with the PI fraction having a high content of α-solanine and solanidine, while the patatin fraction has low values. This difference suggests either a direct association of α-solanine and solanidine with the PI proteins at pH 8 when PI proteins have a neutral-to-positive charge or limited interaction with the anion exchange column used for binding of the patatin proteins. The PI and patatin fractions have been analyzed in a previous study by an HPLC–UV method, with the PI fraction having a content of α-solanine of 932 µg g^−1^ and α-chaconine of 120 µg g^−1^, while the patatin fraction had values of 60 and 41 µg g^−1^ [22]. The two studies show similar trends in regards GA content, but LC–ESI/MS showed a lower concentration than the method based on HPLC, which is likely a result of a more precise measurement by LC–ESI/MS as it only quantifies based on selected ions and hence avoids overestimation by excluding contaminants. In this study, we also found that the PI fraction had a high content of solanidine and that the patatin had a higher solanidine content than the total GA content.

Previous studies have used either UV chromatograms or mass spectrometry analysis using full scan mode. Single ion monitoring is better suited for quantification over scanning across a broad range. As not all *m*/*z* values are recorded, the mass analyzer can acquire more data points per selected ion, which improves sensitivity, as was the case here, by approximately 10-fold. In potato protein powder samples, the current method for extraction does not allow for separation of GA from other compounds in the chromatogram peaks; hence, quantification based on peak area is less precise as contamination likely results in higher values when determining GA concentration. The method also allows for identification of the peaks which likely represents the β-solanine (*m*/*z* 706/722), γ-solanine (*m*/*z* 560), β-chaconine (*m*/*z* 706), and γ-chaconine (*m*/*z* 560).

It is important to be able to distinguish between the different GAs as the toxicity if these are not the same. Friedmann et al. [32] reported that α-chaconine was three times more toxic for frog embryos than α-solanine, and the aglycone solanidine being much less toxic. A published study tested the toxicity of tri-glycoalkaloid, di-glycoalkaloids, and mono-glycoalkaloid in frog embryos and found that toxicity generally decreased following the removal of the sugars of the tri-glycoalkaloid [33]. Furthermore, it was found that the stereochemistry of the di-glycoalkaloids also influenced toxicity.

In vitro studies indicated that 3%–5% of total GA were solubilized during simulated digestion [34]. Furthermore, it has been shown that 5% of α-chaconine and α-solanine were hydrolyzed to other intermediate structures during a 3 h simulated in vitro digestion [25]. A recent study also discovered a bacterial gene cluster involved in deglycosylations of α-chaconine and α-solanine and thereby potentially detoxification of the potato GAs [35].

Pharmacokinetics of orally distributed GA has been performed in human subjects showing differences between α-solanine and α-chaconine. One study estimated the biological half-lives to be 19.1 h and 10.7 h for α-chaconine and α-solanine, respectively. The aglycon solanidine appeared in blood after 4–8 h suggestion in vivo digestion of the two GA, the intermediate structures, mono- and diglycosides, were however not detected [36]. Another study reported half-lives of α-chaconine and α-solanine to be 44 and 21 h, respectively [37]. Both studies found that the serum GA concentration did not returned to baseline after 24 h, suggesting possible accumulation if GA-containing meals are ingested regularly.

In animal studies, it has been shown that orally administered GAs are less toxic than intraperitoneal administration due to poor absorption in the gut. In mice, LD_50_ (milligrams per kilogram of body weight) have been reported to be 23 for α-chaconine, 34 for α-solanine, 500 for solanidine, and >1000 for α-solanine when given orally [38].

Studies have shown that the ratio between α-solanine and α-chaconine is important for toxicity and a mixture of the two alkaloids has a synergistic effect. A 1:1 ratio was reported to show the highest cytotoxic effect in three rat cell studies [39]. A study also reported a synergistic toxic effect of mixing α-solanine and α-chaconine when tested in frog embryos [40], while another study reported a decreased toxicological effects on hamsters fed diets with a reduced α-solanine to α-chaconine ratio [41]. These studies show the importance of reliable quantification of both tri-GAs and aglycons.

## 4. Conclusions

This study reports on the development of a precise method for quantitative measurement of GA in potato protein powder of different purity intended for either food or feed purposes by the industry. The strength of this method is that it can detect and quantify very low levels of GA and their aglycon and hence use small sample sizes. From the work, we also indicated the detection of the hydrolysis product, which could add to the toxicity of the protein product. Our results show that GA is mostly extracted with the PI fraction and that washing can lower the GA content in potato protein powder to an acceptable range for human consumption.

## Figures and Tables

**Table 1:** Ions used in single ion monitoring for quantification of glycoalkaloids (GAs) by LC-ESI/MS.

Compound	[M+H]+	Ions (*m*/*z*)	Peak usage
α-solanine	868.4	868.4	**Target Ion**
		560.4	Qualifier Ion
		398.2	Qualifier Ion
α-chaconine	852.4	852.4	**Target Ion**
		560.4	Qualifier Ion
		398.2	Qualifier Ion
Solanidine	398.2	398.2	**Target Ion**
Tomatine (ISTD)	1034.4	1034.4	Qualifier Ion
		576.4	**Target Ion**
		416.2	Qualifier Ion

**Table 2:** Validation of linearity, limit of detection (LOD), limit of quantification (LOQ), and recovery for individual compounds.

Compound	Linearity (R^2^)	LOD (µg mL^−1^)	LOQ (µg mL^−1^)	Recovery	
				0.06 µg mL^−1^	2 µg mL^−1^
α-Solanine	0.994	0.012	0.03	81.6%	106.4%
α-Chaconine	0.996	0.011	0.03	82.7%	101.5%
Solanidine	0.997	0.003	0.01	90.3%	83.9% ^a^

^a^ 0.6 µg g^−1^ recovery.

**Table 3:** Intra- and inter-day precision and accuracy of α-solanine, α-chaconine, and solanidine standards.

Concentration (µg mL^−1^)	Intra-Day Precision (% CV, *n* = 6)	Intra-Day Accuracy (%, *n* = 6)	Inter-Day Precision (% CV, *n* = 4)	Inter-Day Accuracy (%, *n* = 4)
**α-Solanine**				
0.03	8.07	112.41	1.76	128.81
0.1	8.15	104.01	0.70	108.21
0.3	8.58	103.26	0.12	105.62
1	8.04	88.41	6.93	85.88
3	5.62	101.24	6.77	101.46
**α-Chaconine**				
0.03	7.04	114.33	8.89	79.93
0.1	5.26	99.68	10.53	89.34
0.3	7.12	101.80	8.47	107.85
1	6.45	82.26	10.71	99.42
3	4.26	95.76	4.90	107.96
**Solanidine**				
0.01	2.96	85.97	5.49	89.93
0.03	4.30	87.48	6.14	83.41
0.1	4.50	86.38	4.23	102.35
0.3	4.74	111.89	2.92	106.34
1	5.98	99.09	0.65	99.43

**Table 4:** Glycoalkaloid content in different potato protein isolates (means ± standard deviation, *n* = 6).

			α-Solanine		α-Chaconine		Solanidine	
Sample	Treatment	Total GA	µg g^−1^ (ppm)	%CV	µg g^−1^ (ppm)	%CV	µg g^−1^ (ppm)	%CV
Feed-grade protein	Early ^a^	2061.7 ± 94.9	782.7 ± 40.0	5.1	1279.0 ± 54.9	9.8	38.1 ± 4.4	11.5
	Mid ^a^	2456.8 ± 47.8 *	905.0 ± 23.3 *	2.57	1551.82 ± 24.5 *	1.6	95.6 ± 7.4 *	7.7
	Late ^a^	3188.5 ± 303.0 ***	1129.0±101.9 ***	9.0	2059.5 ± 201.1 ***	9.8	161.2 ± 26.6 ***	16.5
Food-grade protein ^b^	High ^b^	316.0 ± 41.3	118.4 ± 16.7	14.1	197.6± 24.6	10.4	131.3 ± 21.4	16.4
	Low ^b^	28.9 ± 2.3 ***	10.9 ± 1.0 ***	8.7	18.0± 1.3 ***	7.4	41.9 ± 7.4 ***	15.7
PI^c^ fraction	IEX chromatography ^d^	641.1 ± 17.9	606.0 ± 16.3	2.7	35.1 ± 1.6	4.6	549.3 ± 100.3	18.3
Patatin fraction	IEX chromatography ^d^	50.6 ± 3.5 ***	43.2 ± 3.4 ***	7.8	7.4 ± 0.1 ***	1.2	69.6 ± 4.6 ***	6.6

^a^ Protein produced from potatoes at different time point between September–January. ^b^ Potato protein washed similar ways with water but using high or low ratio of water to protein. ^c^ Protease inhibitors. ^d^ Isolated by ion-exchange chromatography [22]. Asterisks show statistical significant differences between samples (* *p* < 0.05; *** *p* < 0.001).
